# Supplementary figures and images for: Development of monoclonal antibody-based blocking ELISA for detecting SARS-CoV-2 exposure in animals
Source: mSphere. 2023 Jul 6;8(4):e00067-23. doi: 10.1128/msphere.00067-23 (PMC10449516; doi:10.1128/msphere.00067-23)

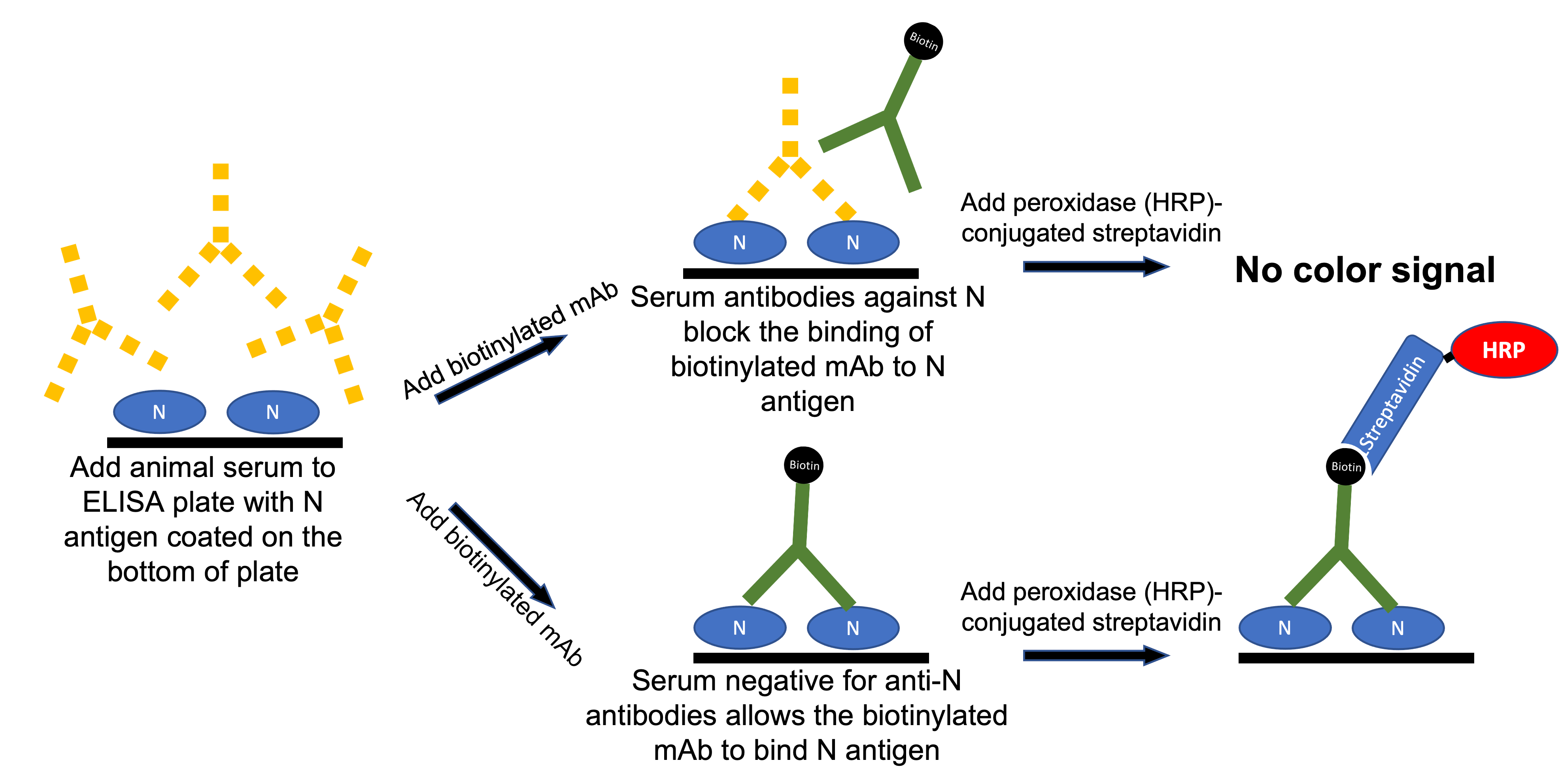

Supplement: Figure S1 — Design of mAb-based bELISA. [file msphere.00067-23-s0001.tif]
