# Supplementary material for: Development of monoclonal antibody-based blocking ELISA for detecting SARS-CoV-2 exposure in animals
Source: mSphere. 2023 Jul 6;8(4):e00067-23. doi: 10.1128/msphere.00067-23 (PMC10449516; doi:10.1128/msphere.00067-23)
Supplement: Supplemental Legend — Legend for Fig. S1. [file msphere.00067-23-s0002.docx]

**Figure S1. Design of monoclonal antibody (mAb)-based blocking ELISA for detecting SARS-CoV-2 exposure in animals.** SARS-CoV-2 antigen is coated onto the bottom of an ELISA plate. If there is presence of anti-N antibodies in the animal serum, they will bind to the N antigen and block the binding of biotinylated anti-N mAb to the N antigen. The mAb will be washed away and no color signal will be developed in the subsequent steps. If there is no anti-N antibodies present in the animal serum, the biotinylated anti-N mAb will bind to the N antigen, then the HRP-conjugated streptavidin will be added and bind to the biotin that conjugated to mAb. HRP substrate will be added to develop the color signal.
